# D-Dimer Levels before HIV Seroconversion Remain Elevated Even after Viral Suppression and Are Associated with an Increased Risk of Non-AIDS Events

**DOI:** 10.1371/journal.pone.0152588

**Published:** 2016-04-18

**Authors:** Matthew S. Freiberg, Ionut Bebu, Russell Tracy, Kaku So-Armah, Jason Okulicz, Anuradha Ganesan, Adam Armstrong, Thomas O’Bryan, David Rimland, Amy C. Justice, Brian K. Agan, Infectious Disease Clinical Research Program HIV Working Group

**Affiliations:** 1 Division of Cardiovascular Medicine, Vanderbilt University School of Medicine and Geriatric Research, Education, and Clinical Center, Veterans Affairs Tennessee Valley Healthcare System, Nashville, TN, United States of America; 2 Infectious Disease Clinical Research Program (IDCRP), Department of Preventive Medicine and Biostatistics, Uniformed Services University of the Health Sciences, Bethesda, MD, United States of America; 3 Infectious Disease Service, San Antonio Military Medical Center, San Antonio, TX, United States of America; 4 Infectious Disease Clinic, Walter Reed National Military Medical Center, Bethesda, MD, United States of America; 5 Department of Pathology, University of Vermont College of Medicine, Burlington, VT, United States of America; 6 The Biostatistics Center, Milken Institute School of Public Health, The George Washington University, Rockville, MD, United States of America; 7 U.S. Naval Medical Research Unit No. 6 Peru, Lima, Peru; 8 Division of General Internal Medicine, Boston University, Boston, MA, United States of America; 9 Division of Infectious Diseases, Department of Medicine, Emory University School of Medicine and VA Medical Center, Atlanta, Georgia, United States of America; 10 Section of General Internal Medicine, Department of Medicine, Yale University School of Medicine, and VA Connecticut Healthcare System West Haven affiliation, New Haven, CT, United States of America; 11 Henry M. Jackson Foundation for the Advancement of Military Medicine, Bethesda, MD, United States of America; University of Pittsburgh Center for Vaccine Research, UNITED STATES

## Abstract

The mechanism underlying the excess risk of non-AIDS diseases among HIV infected people is unclear. HIV associated inflammation/hypercoagulability likely plays a role. While antiretroviral therapy (ART) may return this process to pre-HIV levels, this has not been directly demonstrated. We analyzed data/specimens on 249 HIV+ participants from the US Military HIV Natural History Study, a prospective, multicenter observational cohort of >5600 active duty military personnel and beneficiaries living with HIV. We used stored blood specimens to measure D-dimer and Interleukin-6 (IL-6) at three time points: pre-HIV seroconversion, ≥6 months post-HIV seroconversion but prior to ART initiation, and ≥6 months post-ART with documented HIV viral suppression on two successive evaluations. We evaluated the changes in biomarker levels between time points, and the association between these biomarker changes and future non-AIDS events. During a median follow-up of 3.7 years, there were 28 incident non-AIDS diseases. At ART initiation, the median CD4 count was 361cells/mm^3^; median duration of documented HIV infection 392 days; median time on ART was 354 days. Adjusted mean percent increase in D-dimer levels from pre-seroconversion to post-ART was 75.1% (95% confidence interval 24.6–148.0, p = 0.002). This increase in D-dimer was associated with a significant 22% increase risk of future non-AIDS events (p = 0.03). Changes in IL-6 levels across time points were small and not associated with future non-AIDS events. In conclusion, ART initiation and HIV viral suppression does not eliminate HIV associated elevation in D-dimer levels. This residual pathology is associated with an increased risk of future non-AIDS diseases.

## Introduction

HIV infected (HIV+) people have an excess risk of non-AIDS diseases[1] as compared to uninfected people that is not explained by antiretroviral therapy (ART) toxicity or the presence of risk factors.[2, 3] Although the mechanism(s) underlying this excess risk of non-AIDS diseases (e.g., cardiovascular disease and cancer) is not known, inflammation/hypercoagulability is thought to play an important role.[4, 5] A recent study reported that immediate initiation of ART in asymptomatic HIV+ people with high CD4 cell counts reduces the risk of non-AIDS diseases.[6] However, it is unclear whether this strategy eliminates the excess risk of non-AIDS diseases, in part, because we do not know if this strategy returns HIV associated levels of inflammation/hypercoagulability to pre-HIV seroconversion levels. If HIV+ people have residual inflammation/hypercoagulability (i.e., levels of inflammation/hypercoagulability after viral suppression are higher than levels pre-HIV seroconversion) after achieving viral suppression with ART and if this residual pathology were associated with an increased risk of future non-AIDS diseases, then screening, prevention, and management strategies, above and beyond immediate initiation of ART and viral suppression, would be needed.

To answer this question, we first examined participants from the U.S. Military HIV Natural History Study (NHS) to assess the longitudinal changes in biomarkers of inflammation/hypercoagulability beginning before HIV seroconversion through ART initiation and HIV viral suppression. Next we examined the longitudinal changes in these biomarkers and their association with two outcomes: (1) future non-AIDS events and (2) a composite endpoint that includes future non-AIDS events, AIDS events, and death. We studied IL-6, a marker of inflammation that appears later in the inflammatory cascade, as well as D-dimer, a marker of inflammation and hypercoagulation, both of which have been associated with HIV-related mortality as well as morbidity.[7]

## Methods

### Population and Study Design

The U.S. Military HIV NHS is a prospective observational cohort of >5600 HIV+ active duty military members, dependents, and beneficiaries followed in the military healthcare system since 1986.[8] The Uniformed Services University of the Health Sciences IRB and participating NHS study sites IRBs approved this study. All subjects provided written informed consent. Active duty personnel are screened for HIV approximately every 2 years. NHS study visits occur approximately every six months and include a research coordinator interview, physician visit, and medical record review and biospecimen collection.

For this investigation, we selected participants with an interval of <4 years from last HIV negative to first HIV positive tests (ELISA screen confirmed by repeat ELISA and Western blot when positive). HIV seroconversion is estimated as the midpoint of this window. Eligible participants were on ART for ≥6 months with HIV-1 RNA suppression on at least two successive measurements, and had serum samples available at each of the three time points: before HIV infection; after HIV, but before ART initiation; and post-ART (S1 Fig). Having serum available for each subject at all three time points was essential to allow individuals to serve as their own controls. Suppression was defined as a HIV-1 RNA <50 copies/mL except for 41 participants for whom a longer calendar period between time points included changes in assay sensitivity. For these 41 participants, undetectable viremia was defined as HIV-1 RNA<400 copies/mL at one of the two time points. To reduce confounding, participants had no hepatitis B or C, liver or cardiovascular disease, diabetes, malignancy, inflammatory conditions, or use of steroids prior to the post-ART time point. ART was initiated by individual providers based on treatment guidelines and patient preferences.

### Specimens

Specimen time points were defined as follows: ‘pre-HIV’ (time point 1, TP1)–the latest available sample at or prior to the last documented negative HIV test; ‘pre-ART’ (TP2)–the earliest available sample at least six months after estimated HIV seroconversion and three months after the first HIV positive test but before ART initiation; and ‘post-ART’ (TP3)–the earliest available sample at least six months after ART initiation with viral suppression as above. This sampling strategy was designed *a priori* to: 1) maximize the number of eligible subjects, 2) confine the samples to a limited time span to minimize confounding due to incident comorbidity prior to TP3, and 3) ensure the samples were representative of the stage of disease being evaluated. Pre-HIV specimens were obtained from the DoD Serum Repository[9] and post-HIV specimens from the NHS repository. In the DoD HIV screening program, serum samples remaining after HIV testing are stored at -30°C in a central facility. NHS subjects have blood samples stored at -80°C. For this analysis, serum from the NHS was used to provide comparability to the pre-HIV time point serum from the DoD Serum Repository.

### Data Collection

We extracted participant data from the NHS database including demographics, medical history based on participant interview and medical record review (comorbidities and substance abuse), medication use, HIV history, clinical parameters (height, weight, and blood pressure), laboratory parameters (e.g., CD4 and HIV-1 RNA). Illicit drug use was not assessed, but is low in this population.[10] Self-reported smoking was dichotomized as smoker or non-smoker. At risk alcohol use was defined using standard definitions.[11] Variables associated with exclusion criteria were used to verify subject sampling and as outcomes after TP3. Incident non-AIDS diseases events were new medical diagnosis after TP3 and included diabetes mellitus (n = 14), non-AIDS malignancy (n = 7), asthma (n = 3), acute myocardial infarction (n = 2), peripheral artery disease (n = 1), and cirrhosis (n = 1). These diseases were selected because of their association with HIV and inflammation/coagulation.[12–15] We also specified a composite endpoint including non-AIDS diseases, AIDS (1993 CDC-revised; n = 4), and death (n = 4). Participant self-reported diagnoses were confirmed from physician interview and review of the participant’s medical record.

### Laboratory assays

Standard clinical laboratory assays were done locally in batched analysis. IL-6 and D-dimer were measured at the University of Vermont using assay methods that have been used extensively in HIV research.[4, 5, 16] IL-6 was measured by high-sensitivity ELISA (Quantikine HS Human IL-6 Immunoassay; R&D Systems, Minneapolis, MN). The lower limit of detection (LOD) was 0.16 pg/mL; the coefficient of variation (CV) in this study was 15.4%. D-dimer was measured using an immuno-turbidimetric assay (Liatest D-DI; Diagnostica Stago, Parsippany, NJ) on a Sta-R automated analyzer (Diagnostica Stago, Parsippany, NJ). The lower LOD was 0.02 μg/mL, and the average CV was 5.1%. In addition, since neither EDTA- nor citrate-plasma, the usual sample types for D-dimer analyses, were available for this study we validated the measurement of D-dimer on serum samples by analyzing matched serum and EDTA plasma samples from 20 individuals. Linear regression revealed the following relationship: (D-dimer_serum_) = 1.008 (D-dimer_EDTA_) + 0.27; R = 0.967.

### Statistical Analysis

Continuous variables were described using medians (interquartile ranges), and means (standard deviations), and categorical variables as percentages. The t-test or the Mann-Whitney test was used for continuous variables, while chi-squared tests or Fisher exact tests for discrete variables. Due to non-normal distributions, the biomarker values were analyzed on the log scale in models below.

We evaluated change in biomarker levels at pairwise time points using the Wilcoxon signed rank test (e.g., TP1 vs. TP3). The percent increase in biomarker values, for example from TP1 to TP3, was calculated using the formula (TP3-TP1)/TP1)*100. We used linear models to adjust for confounders. To investigate the relationship between the change in biomarker value and the baseline (pre-HIV) value, the biomarker value at the first time point was also included in the model. A negative coefficient suggests that subjects with lower baseline biomarker value had a higher increase at the subsequent time point. Sensitivity analyses were performed to assess the robustness of the results. While D-Dimer values below the detection limit 0.02 (n = 20) were set to 0.01 for main analyses, in sensitivity analyses, we excluded them. A similar approach was taken for IL-6 with the first sensitivity analysis excluding the highest and lowest 5%, and the second setting values above the upper 1^st^ and 5^th^ percentile to the corresponding 1^st^ and 5^th^ percentile values (i.e. winsorizing). Nonparametric bootstrap was used for assessing the sensitivity of the results with respect to the parametric assumptions in the linear models.[17] No significant differences in results were observed using these different analytic approaches.

We used adjusted Cox proportional hazards models to investigate the association between the change in biomarker levels across time points and the risk of non-AIDS diseases in a time-to-event analysis including all subjects. For this analysis the hazard ratio (HR) corresponds to one unit change in the biomarker value on the log scale. To calculate the HR for any percent change in the biomarker level for the risk of non-AIDS diseases and composite events (denoted by HR(x)) we used the formula HR(x) = HR^log (x+1)^ where HR is the hazard ratio of the biomarker on the log scale. The start time was the post-ART specimen (TP3); the outcome was time to the first event. Models were constructed separately for the two outcomes (non-AIDS diseases and the composite clinical events). Because smoking and alcohol use have only been routinely assessed in the cohort since 2006, missing values for smoking and alcohol use were included as a separate category, ‘missing’, in order to maintain model robustness. All tests were two-sided, and p-values less than 0.05 were considered statistically significant.

## Results

Participants in the NHS prior to HIV seroconversion were young, mostly male, and nearly half Caucasian (Table 1). On average, at the time of HIV infection, these participants were normotensive, without hypercholesterolemia and had normal levels of serum hepatic transaminases, hemoglobin, and creatinine (Table 2). The median BMI was 26 kg/m^2^ and no participants had diabetes at study entry. Of those reporting smoking and alcohol status, the majority were non-smokers and not at-risk drinkers. ART use was non-nucleoside reverse transcriptase inhibitor based for 65.6%, protease inhibitor for 28.8%, and integrase inhibitor based/other for 5.6% (data not shown).

**Table 1:** Baseline and time point variables description (N = 249).

	Median (IQR) or N (%)
**Age (TP1)**	27 (23,32)
**Gender**	
Male	244 (98%)
Female	5 (2%)
**Race/Ethnicity**	
Caucasian	113 (45%)
African American	93 (37%)
Hispanic/Other	43 (17%)
**Alcohol**	
At Risk	69 (28%)
Not at Risk	123 (50%)
No Use	16 (7%)
Missing	41 (17%)
**Smoking**	
Yes	57 (23%)
No	97 (39%)
Missing	95 (39%)
**D-Dimer levels (ug/mL)**^1^^,^^2^	
TP1	0.17 (0.09,0.31)
TP2	0.44 (0.23,0.89)
TP3	0.24 (0.16,0.41)
**IL-6 levels (pg/mL)**^1^	
TP1	1.84 (1.07,5.60)
TP2	1.89 (1.29,3.30)
TP3	1.67 (1.11,3.04)
**Specimen Intervals (yrs)**	
TP1 to estimated seroconversion	0.9 (0.56,1.42)
TP1 to 1^st^ documented HIV positive test	1.7 (1.1,2.3)
Seroconversion to TP2	1.91 (1.10,3.54)
1^st^ documented HIV positive test to TP2	0.98 (0.8, 1.2)
ART initiation to TP3	0.97 (0.74,1.1)

^1^Not log transformed

^2^p<0.01 for all pairwise comparisons between Time point (TP) 1,2,3

abbreviations: TP# = time point; IQR = interquartile range; ART = antiretroviral therapy ‘pre-HIV’ (time point 1, TP1)–the latest available sample at or prior to the last documented negative HIV test; ‘pre-ART’ (TP2)–the earliest available sample at least six months after estimated HIV seroconversion and three months after the first HIV positive test but before ART initiation; and ‘post-ART’ (TP3)–the earliest available sample at least six months after ART initiation with viral suppression as above

**Table 2:** Clinical and Laboratory Parameters while HIV+ (N = 249).

Clinical Parameters	TP2 median (IQR)	TP3 median (IQR)	p-value
Body Mass Index kg/m^2^	26 (24,29)	26 (24,29)	0.28
Systolic Blood Pressure (mm Hg)	126 (117,134)	124 (118,133)	0.26
Diastolic Blood Pressure (mm Hg)	78 (72,82)	80 (74,86)	0.85
**Laboratory Parameters**			
CD4 count (cells/mm^3^)	361 (288,455)	549 (441,711)	<0.001
HIV viral copies (log10)	4.53 (4.00 4.78)	1.7 (1.7,1.7)	<0.001
Hemoglobin (g/dL)	15 (14,15)	15 (14,16)	0.002
Creatinine (mg/dL)	1.0 (0.9,1.1)	1.0 (0.9,1.0)	0.004
Alanine Aminotransferase (ALT, U/L))	31 (22,45)	30 (23,41)	0.21
Aspartate Aminotransferase (AST, U/L))	28 (24,37)	26 (22,32)	0.002
Total Cholesterol (mg/dL)	163 (145,188)	183 (156,215)	<0.001
Low Density Lipoprotein (LDL) Cholesterol (mg/dL)	98 (79,116)	107 (89,136)	0.002
Triglycerides (mg/dL)	105 (70,160)	139 (84,200)	<0.001

Abbreviations: TP# = time point; IQR = interquartile range

Using the estimated seroconversion date, the average duration of HIV infection at TP2 was 1.91 years, and the average interval from ART initiation to >6 months viral suppression (TP3) was 354 days. Before ART initiation (TP2), median CD4 cell count was 361 cells/mm^3^ and log_10_ viral load was 4.4. At viral suppression (TP3) after ART initiation, the median CD4 cell count was 549 cells/mm^3^. The median CD4 nadir was 315 cells/mm^3^. Compared across time points, changes in laboratory covariates involving hemoglobin, creatinine, AST, ALT, and cholesterol values did not vary clinically from TP2 to TP3 (Table 2). In fact, at the time of HIV viral suppression, total cholesterol was at “desirable” levels, LDL cholesterol was “near optimal,” and serum triglycerides were “normal” as per National Cholesterol Education Program/Adult Treatment Panel

**Fig 1:**
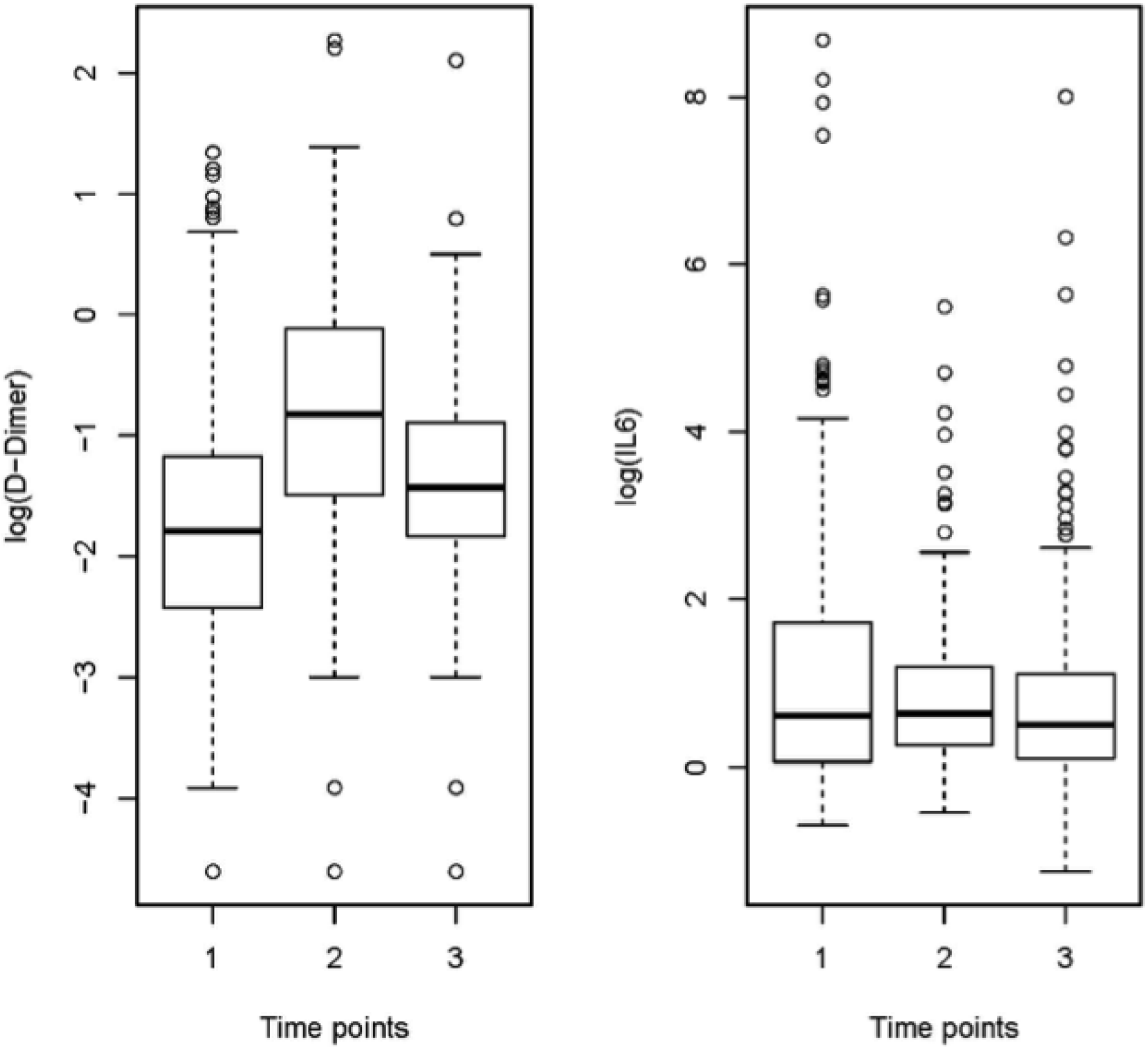
Population mean D-Dimer and IL-6 at the three time points. Box and whisker plot describes median (line inside of box), lower and upper quartiles (bottom and top of box), minimum (horizontal line below vertical dashed line), maximum (horizontal line above vertical dashed line) and outliers (open circles) for D-dimer and IL-6 distributions.

In unadjusted analyses, post-ART initiation (TP3) population median D-dimer levels were significantly higher than pre-HIV seroconversion levels (TP1), showing evidence of a “residual” elevation in D-dimer (Fig 1). 59% of subjects had TP3 D-dimer levels higher than baseline (TP1), while 41% of subjects had levels at or below baseline (TP1). After ART initiation and viral suppression (TP3), D-dimer levels were significantly lower compared to D-dimer levels at TP2 (Table 1). Pre-ART initiation (TP2) D-dimer levels were significantly higher than levels at TP1 and TP3 (p<0.01 for both). By comparison, the differences in IL-6 levels across the three time points were not statistically different (Fig 1, p>0.05 for all).

After adjusting for covariates, the percent increase in D-dimer from TP1 to TP3 was 75.1% (P = 0.002, Table 3). Restricting the sample to never smokers (n = 98), had little effect on this increase (70.0%, p<0.01). In contrast, levels of IL-6 did not differ across time points (Table 3). Clinical and laboratory measures of smoking, at risk alcohol consumption, blood pressure, anemia, kidney and hepatic function, and cholesterol did not differ between participants with or without residual biomarker elevations (Table 4).

**Table 3:** Multivariate analysis showing mean percent change in biomarker levels from pre-HIV to post-HAART*.

	D-Dimer			IL-6		
	Estimate	95% CI	P-value	Estimate	95% CI	P-value
Biomarker change (from TP1 to TP3)**	75%	(24.6–148)	0.002	2%	(-29.2,33.1)	0.90

*adjusted for age, race, smoking, blood pressure, body mass index, time from estimated seroconversion to HIV+, time from HIV+ to ART initiation, biomarker at TP1, CD4, hemoglobin, creatinine, and lipids; CI = 95% confidence interval

**biomarker values were log transformed and the percent change in biomarker values is shown [100(e^β^-1)]

Abbreviations: TP# = time point; CI = confidence interval

**Table 4:** Characteristics of participants who had and did not have residual elevations in biomarker levels of IL-6 and D-dimer.

	D-dimer				IL-6			
	Total	Return to baseline levels		P-value	Total^a^	Return to baseline levels		P-value
		Yes	No			Yes	No	
**Age (years)**	31 (28,37)	32 (28,37)	31 (28,37)	1.0	31 (28,37)	31 (27,36)	32 (29,38)	0.05
**Ethnicity (%)**								
**Caucasian (n)**	47	41	47	0.60	45	42	49	0.45
**African American (n)**	39	41	35		37	38	36	
**Hispanic/Other (n)**	18	18	18		17	20	15	
**Gender Male (%)**	98	98	98	1.00	98	96	100	0.06
**Alcohol At Risk (%)**	29	26	30	0.47	28	32	22	0.34
**Smoking Yes (%)**	24	24	23	0.75	23	21	25	0.73
**Body mass index (kg/m**^**2**^**)**^1^	26 (24,29)	27 (24,30)	26 (24,29)	0.02	26 (24,29)	26 (24,29)	26 (24,29)	0.99
**Systolic blood pressure (mm Hg)**^1^	124 (118,133)	124 (120,133)	124 (118,132)	0.61	124 (118,133)	123 (118,132)	126 (119,133)	0.29
**Diastolic blood pressure (mm Hg)**^1^	80 (74,86)	80 (75,86)	80 (73,86)	0.83	80 (74,86)	79 (74,84)	81 (75,87)	0.12
**CD4 cell count (cells/mm**^**3**^**)**^1^	549 (441,711)	532 (422,683)	567 (454,726)	0.51	549 (441,711)	540 (434,718)	552 (448,706)	0.65
**Viral load Copies**^1^^,^^2^ **(log**_**10**_**)**	1.7 (1.7,1.7)	1.7 (1.7,1.7)	1.7 (1.7,1.7)	0.18	1.7 (1.7,1.7)	1.7 (1.7,1.7)	1.7 (1.7,1.7)	0.07
**Hemoglobin (g/dL)**^1^	15 (14,16)	15 (14,16)	15 (14,16)	0.63	15 (14,16)	15 (14,16)	15 (14,16)	0.41
**Serum Creatinine (mg/dL)**^1^	1.0 (0.9,1)	1.0 (0.9,1)	1.0 (0.9,1.1)	0.59	1.0 (0.9,1)	0.9 (0.9,1)	1.0 (0.9,1.1)	0.11
**ALT (U/L)**^1^	30 (23,41)	30 (23,41)	30 (23,40)	0.28	30 (23,41)	30 (22,40)	30 (24,42)	0.29
**AST (U/L)**^1^	26 (22,32)	26 (22,32)	26 (22,32)	0.36	26 (22,32)	26 (22,32)	26 (22,32)	0.29
**Serum Total Cholesterol (mg/dL)**^1^	183 (156,215)	183 (155,213)	180 (158,215)	0.23	183 (156,215)	180 (155,208)	190 (160,220)	0.244
**LDL cholesterol (mg/dL)**^1^	107 (89,136)	108 (88,134)	106 (89,136)	0.62	107 (89,136)	106 (88,134)	109 (90,138)	0.805
**Serum Triglycerides (mg/dL)**^1^	139 (84,200)	138 (86,222)	132 (78,184)	0.11	139 (84,200)	116 (77,185)	152 (90,225)	0.425

^a^median (IQR) unless otherwise stated

^1^at TP3

^2^log 1.7 = <50 copies/mL

Abbreviations: TP = time point; IQR = interquartile range; ALT = Alanine Aminotransferase; AST = Aspartate Aminotransferase; LDL = Low Density Lipoprotein

During a median follow up time of 3.7 years (1133 person years total), there were 28 incident non-AIDS diseases and 36 incident composite events (rates (95% confidence interval (CI)) of 24 (16–38) and 30 (21–42), per 1000 person years, respectively). After adjusting for confounders, a one unit change in the log D-dimer was associated with a 43% and a 35% increase risk of future non-AIDS diseases and composite events, respectively (Table 5). Based on results from Table 3, a 75.1% increase in D dimer levels from TP1 to TP3 is associated with a 22% increase in the risk of future non-AIDS diseases (p = 0.03) and an 18% increase risk in future composite events (p = 0.03). Analyses involving log IL-6 in adjusted models did not reach statistical significance (Table 5).

**Table 5:** Cox Models for Time to event (non-AIDS diseases and Composite Event) by Biomarker*.

	D-Dimer			IL-6		
	HR	95% CI	P-value	HR	95% CI	P-value
**NON-AIDS DISEASES**						
Change in Biomarker**	1.43	(1.04–2.00)	0.03	1.29	(0.73–2.27)	0.38
**Composite Event (non-AIDS diseases, AIDS, death)**						
Change in Biomarker**	1.35	(1.03–1.77)	0.03	1.33	(0.82–2.16)	0.25

*adjusted for age, smoking, body mass index, CD4, hemoglobin, creatinine, lipids, and TP3 biomarker level

**biomarker values were log transformed and the HRs correspond to one unit change in the biomarker value on the log scale. These values can be converted to x-percent change in the biomarker value using the following equation: HR^log(x+1)^; for example, the hazard ratio for non-AIDS associated with 20% change in the D-Dimer value is 1.43^log(1.2)^ = 1.07, with a 95% CI given by (1.01, 1.13).

Abbreviations: TP = time point; HR = hazard ratio; CI = confidence interval

## Discussion

To our knowledge, this is the first large study to examine the longitudinal changes in biomarkers of inflammation/hypercoagulability beginning before HIV seroconversion through ART initiation and HIV viral suppression and their association with future non-AIDS diseases. Our results are three fold: First, that HIV seroconversion is associated with a significant increase in serum D-dimer levels even after adjustment for confounders; second, that adjusted D-dimer levels after ART initiation and HIV viral suppression remain significantly elevated compared to pre-HIV seroconversion levels; and third, that failure to return to pre-HIV levels of D-dimer is significantly associated with an increased risk of future non-AIDS events. Importantly, our study included young, healthy participants with few poor health behaviors who, on average, had ART initiated within 2.7 years of HIV seroconversion and achieved HIV viral suppression within approximately one year of ART initiation; these represent close to “optimal” real-world conditions for people experiencing HIV infection.

The HIV associated inflammation/hypercoagulability state is a product of innate and adaptive cellular immune activation, and altered coagulation with each of these processes likely impacting the other two.[18] This means that alterations in D-dimer, for example, may not simply reflect changes in coagulation status but more likely reflect changes to the immune system more broadly. Our results demonstrating that HIV infection is associated with an increase in D-dimer levels and that D-dimer levels are reduced after treatment are consistent with several earlier studies.[12, 19–21] Similarly, our results are also consistent with earlier reports showing elevated D-dimer levels are associated with incident non-AIDS diseases among HIV+ people.[5, 22, 23]

Unlike prior studies, however, our results are the first to provide direct evidence that rapid HIV diagnosis after seroconversion, ART initiation, and HIV viral suppression does not eliminate, on average, the elevations in D-dimer associated with HIV seroconversion. Moreover, our findings provide support for the emerging hypothesis that this residual pathology is a driver of future non-AIDS diseases. Unlike with D-dimer, we saw no significant increase in IL-6 levels with HIV seroconversion; nor a significant decrease in IL-6 levels with subsequent ART initiation and HIV viral load suppression. While the reason for this finding is not clear, prior studies involving MACS and WIHS study participants also demonstrate little change in IL-6 biomarker levels before and after ART initiation with HIV viral suppression.[19, 20] Additionally, IL-6 biomarker levels may have been attenuated among our study participants because they were young and lacked comorbid diseases that are typically associated with biomarkers like IL-6.[12, 24, 25]

Our results have important clinical implications for HIV+ people and their providers. For HIV+ people, successful ART and viral suppression does not mean that the inflammation/hypercoagulability state associated with HIV is eliminated. HIV+ people are now potentially living decades longer[26] and, for some, also living with other proinflammatory conditions like viral co-infections and substance use.[27] This means that the risk of future non-AIDS diseases, like cardiovascular disease and cancer, are central health issues facing the HIV community.[28] Presently, there is no strategy to identify which individual patients will have this “residual” inflammation/hypercoagulability state because few patients have biomarker levels measured prior to HIV infection. However, our results taken together with longitudinal data from the SMART study[2, 6] and others[20] should prompt the development of population level screening, prevention, and management strategies to reduce the risk of non-AIDS diseases, above and beyond achieving HIV viral suppression, even among healthy HIV+ people. Whether interventions targeting the inflammation/hypercoagulability state in HIV virally-suppressed people would lower the risk of non-AIDS diseases remains unknown but could be answered through interventional trials.

This study has limitations. First, our findings need to be replicated in women. Second, this study examined only two biomarkers. We selected IL-6 and D-dimer because elevations in these biomarkers are associated with an increased risk of non-AIDS diseases.[5, 29–31] Others have examined a broader panel of cytokines cross-sectionally[20] and future analyses examining additional biomarkers of cellular activation, inflammation, and altered coagulation would likely enhance our understanding of the biologic processes.[32] Third, while our sample size precluded analyses examining the association between biomarker changes and specific non-AIDS events, this study remains the largest of its kind because biospecimens before HIV seroconversion are rare. Fourth as we did not have repeated biomarker measures at longer follow-up time points, we don’t know if additional years of sustained HIV viral suppression would have eliminated residual D-dimer levels for all participants; however this is unlikely given cross-section data of others.[4] Lastly, we cannot eliminate the possibility of unmeasured confounding.

In conclusion, HIV seroconversion is associated with a significant increase in serum D-dimer levels. After ART initiation and HIV viral suppression, D-dimer levels remains significantly elevated compared to pre-HIV seroconversion levels. The residual increase in D-dimer levels is associated with an increased risk of incident non-AIDS events. These findings provide direct evidence that ART initiation and HIV viral suppression does not eliminate elevations in D-dimer associated with HIV seroconversion and suggest that successful ART with viral suppression alone, is inadequate to prevent future non-AIDS events. Future research should focus on screening and management strategies for the prevention of non-AIDS events, even among healthy people achieving HIV viral suppression.

## Supporting Information

S1 Fig Study design showing time points (TP) at which blood specimens were tested for D-dimer and Interleukin-6. (TIFF)

## References

[1] Althoff KN, McGinnis KA, Wyatt CM, Freiberg MS, Gilbert C, Oursler KK, et al Comparison of risk and age at diagnosis of myocardial infarction, end-stage renal disease, and non-AIDS-defining cancer in HIV-infected versus uninfected adults. Clin Infect Dis. 2015;60(4):627–38. 10.1093/cid/ciu869 25362204 PMC4318916

[2] El-Sadr WM, Lundgren JD, Neaton JD, Gordin F, Abrams D, Arduino RC, et al CD4+ count-guided interruption of antiretroviral treatment. The New England journal of medicine. 2006;355(22):2283–96. Epub 2006/12/01. 10.1056/NEJMoa062360 . 17135583

[3] Freiberg MS, Chang CC, Kuller LH, Skanderson M, Lowy E, Kraemer KL, et al HIV infection and the risk of acute myocardial infarction. JAMA internal medicine. 2013;173(8):614–22. 10.1001/jamainternmed.2013.3728 . 23459863 PMC4766798

[4] Neuhaus J, Jacobs DR Jr., Baker JV, Calmy A, Duprez D, La Rosa A, et al Markers of inflammation, coagulation, and renal function are elevated in adults with HIV infection. J Infect Dis. 2010;201(12):1788–95. Epub 2010/05/08. 10.1086/652749 20446848 PMC2872049

[5] Kuller LH, Tracy R, Belloso W, De Wit S, Drummond F, Lane HC, et al Inflammatory and coagulation biomarkers and mortality in patients with HIV infection. PLoS Med. 2008;5(10):e203 Epub 2008/10/24. 08-PLME-RA-0628 [pii] 10.1371/journal.pmed.0050203 18942885 PMC2570418

[6] Group ISS, Lundgren JD, Babiker AG, Gordin F, Emery S, Grund B, et al Initiation of Antiretroviral Therapy in Early Asymptomatic HIV Infection. The New England journal of medicine. 2015;373(9):795–807. 10.1056/NEJMoa1506816 26192873 PMC4569751

[7] Kuller L, Group SS, editors. Elevated Levels of Interleukin-6 and D-dimer Are Associated with an Increased Risk of Death in Patients with HIV. Conference on Retroviruses and Opportunistic Infections; 2008 February 3–6; Boston, Massachusetts.

[8] Marconi VC, Grandits GA, Weintrob AC, Chun H, Landrum ML, Ganesan A, et al Outcomes of highly active antiretroviral therapy in the context of universal access to healthcare: the U.S. Military HIV Natural History Study. AIDS research and therapy. 2010;7:14 10.1186/1742-6405-7-14 20507622 PMC2894737

[9] Rubertone MV, Brundage JF. The Defense Medical Surveillance System and the Department of Defense serum repository: glimpses of the future of public health surveillance. American journal of public health. 2002;92(12):1900–4. Epub 2002/11/28. 12453804 10.2105/ajph.92.12.1900 PMC1447349

[10] Brodine SK, Shaffer RA, Starkey MJ, Tasker SA, Gilcrest JL, Louder MK, et al Drug resistance patterns, genetic subtypes, clinical features, and risk factors in military personnel with HIV-1 seroconversion. Ann Intern Med. 1999;131(7):502–6. Epub 1999/10/03. . 10507958 10.7326/0003-4819-131-7-199910050-00004

[11] Dawson DA, Pulay AJ, Grant BF. A comparison of two single-item screeners for hazardous drinking and alcohol use disorder. Alcoholism, clinical and experimental research. 2010;34(2):364–74. Epub 2009/12/03. 10.1111/j.1530-0277.2009.01098.x . 19951291

[12] Armah KA, McGinnis K, Baker J, Gibert C, Butt AA, Bryant KJ, et al HIV status, burden of comorbid disease, and biomarkers of inflammation, altered coagulation, and monocyte activation. Clin Infect Dis. 2012;55(1):126–36. 10.1093/cid/cis406 22534147 PMC3493182

[13] Betene ADC, De Wit S, Neuhaus J, Palfreeman A, Pepe R, Pankow JS, et al Interleukin-6, high sensitivity C-reactive protein, and the development of type 2 diabetes among HIV-positive patients taking antiretroviral therapy. J Acquir Immune Defic Syndr. 2014;67(5):538–46. 10.1097/QAI.0000000000000354 25393940 PMC4231540

[14] Rincon M, Irvin CG. Role of IL-6 in asthma and other inflammatory pulmonary diseases. Int J Biol Sci. 2012;8(9):1281–90. 10.7150/ijbs.4874 23136556 PMC3491451

[15] Peters L, Neuhaus J, Duprez D, Neaton JD, Tracy R, Klein MB, et al Biomarkers of inflammation, coagulation and microbial translocation in HIV/HCV co-infected patients in the SMART study. J Clin Virol. 2014;60(3):295–300. 10.1016/j.jcv.2014.03.017 . 24793968

[16] Armah KA, Quinn EK, Cheng DM, Tracy RP, Baker JV, Samet JH, et al Human immunodeficiency virus, hepatitis C, and inflammatory biomarkers in individuals with alcohol problems: a cross-sectional study. BMC infectious diseases. 2013;13:399 10.1186/1471-2334-13-399 23987993 PMC3848623

[17] Efron B, Tibshirani RJ. An Introduction to the Bootstrap (Chapman & Hall/CRC Monographs on Statistics & Applied Probability). 1994.

[18] Delvaeye M, Conway EM. Coagulation and innate immune responses: can we view them separately? Blood. 2009;114(12):2367–74. 10.1182/blood-2009-05-199208 . 19584396

[19] Palella FJ Jr., Gange SJ, Benning L, Jacobson L, Kaplan RC, Landay AL, et al Inflammatory biomarkers and abacavir use in the Women's Interagency HIV Study and the Multicenter AIDS Cohort Study. AIDS. 2010;24(11):1657–65. Epub 2010/07/01. 10.1097/QAD.0b013e3283389dfa 00002030-201007170-00006 [pii]. . 20588104 PMC3514460

[20] Wada NI, Jacobson LP, Margolick JB, Breen EC, Macatangay B, Penugonda S, et al The effect of HAART-induced HIV suppression on circulating markers of inflammation and immune activation. AIDS. 2015;29(4):463–71. 10.1097/QAD.0000000000000545 25630041 PMC4311407

[21] Baker JV, Brummel-Ziedins K, Neuhaus J, Duprez D, Cummins N, Dalmau D, et al HIV Replication Alters the Composition of Extrinsic Pathway Coagulation Factors and Increases Thrombin Generation. Journal of the American Heart Association. 2013;2(4):e000264 10.1161/JAHA.113.000264 23896681 PMC3828789

[22] Borges AH, O'Connor JL, Phillips AN, Baker JV, Vjecha MJ, Losso MH, et al Factors associated with D-dimer levels in HIV-infected individuals. PLoS One. 2014;9(3):e90978 10.1371/journal.pone.0090978 24626096 PMC3953205

[23] Nordell AD, McKenna M, Borges AH, Duprez D, Neuhaus J, Neaton JD, et al Severity of cardiovascular disease outcomes among patients with HIV is related to markers of inflammation and coagulation. Journal of the American Heart Association. 2014;3(3):e000844 10.1161/JAHA.114.000844 24870935 PMC4309077

[24] Koethe JR, Dee K, Bian A, Shintani A, Turner M, Bebawy S, et al Circulating interleukin-6, soluble CD14, and other inflammation biomarker levels differ between obese and nonobese HIV-infected adults on antiretroviral therapy. AIDS Res Hum Retroviruses. 2013;29(7):1019–25. 10.1089/AID.2013.0016 23469772 PMC3685684

[25] Fuster D, Tsui JI, Cheng DM, Quinn EK, Armah KA, Nunes D, et al Interleukin-6 Is Associated with Noninvasive Markers of Liver Fibrosis in HIV-Infected Patients with Alcohol Problems. AIDS Res Hum Retroviruses. 2013 Epub 2013/04/23. 10.1089/AID.2012.0348 . 23601055 PMC3715787

[26] Palella FJ Jr., Delaney KM, Moorman AC, Loveless MO, Fuhrer J, Satten GA, et al Declining morbidity and mortality among patients with advanced human immunodeficiency virus infection. HIV Outpatient Study Investigators. The New England journal of medicine. 1998;338(13):853–60. Epub 1998/03/27. 10.1056/NEJM199803263381301 . 9516219

[27] Justice AC, Lasky E, McGinnis KA, Skanderson M, Conigliaro J, Fultz SL, et al Medical disease and alcohol use among veterans with human immunodeficiency infection: A comparison of disease measurement strategies. Med Care. 2006;44(8 Suppl 2):S52–60. 10.1097/01.mlr.0000228003.08925.8c . 16849969

[28] Deeks SG, Lewin SR, Havlir DV. The end of AIDS: HIV infection as a chronic disease. Lancet. 2013;382(9903):1525–33. 10.1016/S0140-6736(13)61809-7 24152939 PMC4058441

[29] Duprez DA, Neuhaus J, Kuller LH, Tracy R, Belloso W, De Wit S, et al Inflammation, coagulation and cardiovascular disease in HIV-infected individuals. PLoS One. 2012;7(9):e44454 10.1371/journal.pone.0044454 22970224 PMC3438173

[30] Wannamethee SG, Whincup PH, Shaper AG, Rumley A, Lennon L, Lowe GD. Circulating inflammatory and hemostatic biomarkers are associated with risk of myocardial infarction and coronary death, but not angina pectoris, in older men. Journal of thrombosis and haemostasis: JTH. 2009;7(10):1605–11. Epub 2009/08/18. 10.1111/j.1538-7836.2009.03574.x 19682232 PMC2810437

[31] Ridker PM, Rifai N, Stampfer MJ, Hennekens CH. Plasma concentration of interleukin-6 and the risk of future myocardial infarction among apparently healthy men. Circulation. 2000;101(15):1767–72. Epub 2000/04/19. . 10769275 10.1161/01.cir.101.15.1767

[32] Deeks SG, Tracy R, Douek DC. Systemic effects of inflammation on health during chronic HIV infection. Immunity. 2013;39(4):633–45. 10.1016/j.immuni.2013.10.001 24138880 PMC4012895

