# Supplementary figures and images for: D-Dimer Levels before HIV Seroconversion Remain Elevated Even after Viral Suppression and Are Associated with an Increased Risk of Non-AIDS Events
Source: PLoS One. 2016 Apr 18;11(4):e0152588. doi: 10.1371/journal.pone.0152588 (PMC4835105; doi:10.1371/journal.pone.0152588)

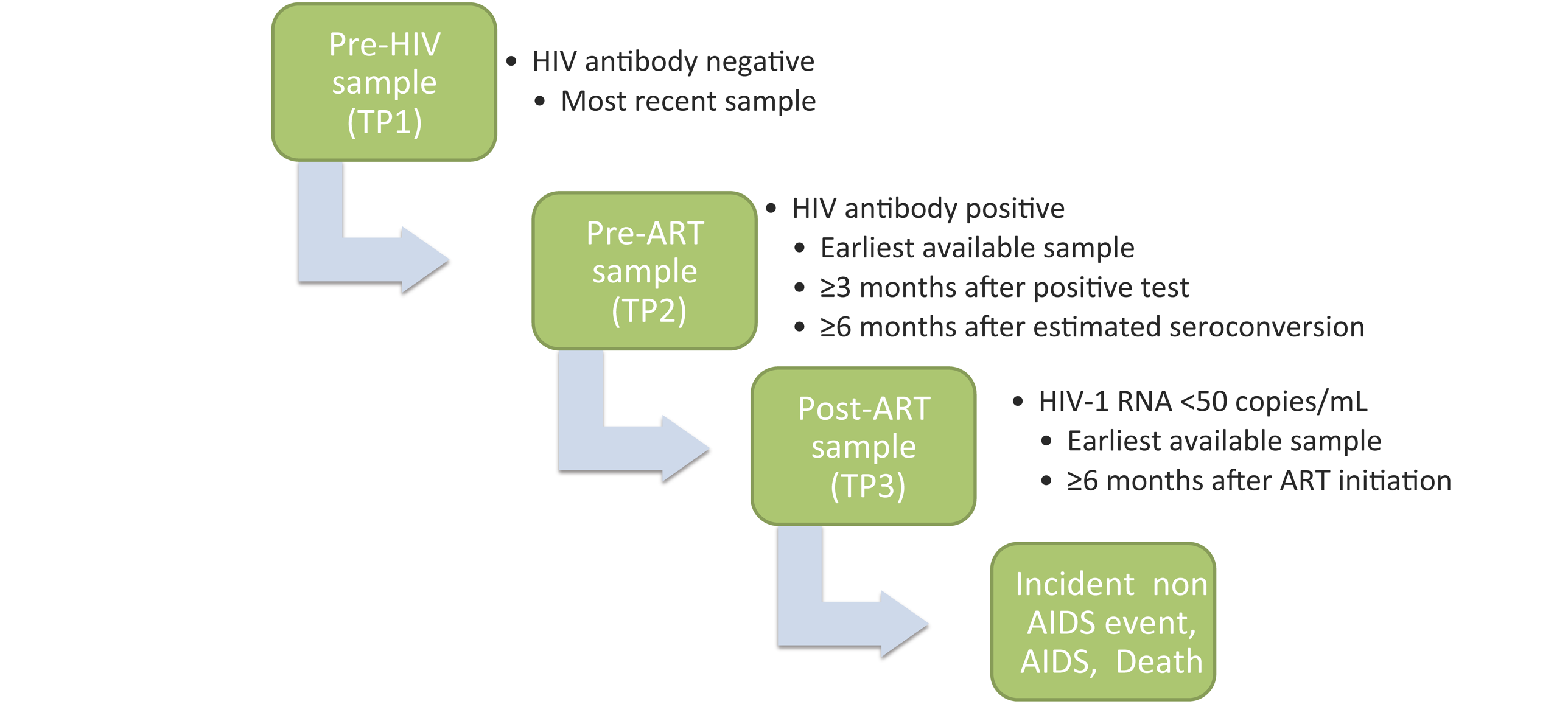

Supplement: S1 Fig — (TIFF) [file pone.0152588.s001.tiff]
